# Chasing a dream against all odds

**DOI:** 10.1186/s12889-022-14130-8

**Published:** 2022-09-15

**Authors:** Eva Vivian, Betty Chewning, Constance Flanagan

**Affiliations:** 1 University of Wisconsin-Madison School of Pharmacy, 777 Highland Avenue, Madison, WI 53705 USA; 2 School of Human Ecology, University of Wisconsin-Madison, Madison, WI 53706 USA

**Keywords:** Poverty, Children, Youth, Resilience, Draw and write, Families, Parents, Draw and write

## Abstract

**Background:**

Youth of color growing up in poverty face many challenges that children from more affluent families never experience. These children often reside in disadvantaged neighborhoods with substandard housing, inadequate medical care, and under resourced schools. This places these children at risk for poor academic achievement, school dropout, abuse and neglect, behavioral and socioemotional problems, and physical health problems. In spite of these risks, some children “beat the odds” and overcome the challenges and adversities in their external contexts. The paper reports the findings of a draw-and-write activity designed to learn the processes whereby protective factors promote resilience from a child’s point of view.

**Methods:**

In this qualitative study, a draw-and-write activity was conducted with a convenience sample of 33 children, (23 females and 10 males of which 10 were Hmong, 11 were Middle Eastern, and 12 were African Americans) .The children were asked to make visual representations of resources (persons or things that, in their view, contribute to their wellbeing.) In depth interviews with a subset of 15 of the children was conducted to discuss the meaning of the images in their drawings. A summative content analysis of the visual and narrative data was performed using a resilience framework.

**Results:**

Regardless of racial/ethnic background, parents, and especially mothers, were the main “person or thing” identified by these children living in poverty as helping them “make it thus far in life.” Ninety seven percent of the participants in this study described their parent(s) as nurturing and supportive, enabling them to overcome obstacles and adversities within their environment.

Forty five percent of participants identified their mother as a key anchor in their life Fifty eight percent of the African American children indicated that their parent(s) encouraged education to escape poverty.

**Conclusion:**

The findings support that families, particularly parents have the strongest influence on supporting the resilience process in a child. These findings were consistent across ethnicity and gender. Families, particularly parents, should be the target of future interventions designed to produce resilient behaviors in youth of color living in poverty.

## Background

Youth of color growing up in poverty face many challenges that children from more affluent families never experience. These children often reside in unsafe neighborhoods where there are high levels of crime, substandard housing, inadequate medical care, and under resourced schools [1]. These children are at greater risk for depression, low self-esteem, poor academic achievement, school dropout, abuse and neglect, behavioral and socioemotional problems, physical health problems, and developmental delays [2].

The stress of living in poverty and struggling to meet daily needs can also impair parenting [2, 3]. Parents living in poverty may experience chronic stress, depression, marital distress and exhibit harsher parenting behaviors, contributing to poor social and emotional outcomes for their children [2]. Parents may have limited education and financial resources reducing their ability to invest in cognitively-stimulating materials and extracurricular educational activities such as sports, music, and dance [1]. Living in homes where there are fewer books to read, and fewer chances for cognitive stimulation may decrease the child’s confidence which, in turn, leads to academic failure [4, 5]. Under-resourced schools in poorer communities often struggle to meet the learning needs of their students and support them in reaching their full potential. Inadequate education contributes to the cycle of poverty by making it more difficult for low-income children to lift themselves and future generations out of poverty [5, 6].

Much of the research conducted in youth of color living in poverty focuses on interventions designed to fix problems associated with exposure to risk [7–10]. While these studies are necessary and useful, they are deficit based and focus on changing behaviors [8]. Researchers who embrace a resilience paradigm acknowledge that despite these risks some youth “beat the odds” and grow up exhibiting positive behaviors [9].

While there is ongoing debate about the formal definition of resilience, there is consensus that resilience is the ability to recover from or adapt to difficult and challenging life circumstances [9–12]. Contrary to popular belief, resilience depends not only on ‘internal grit or drive’ but relationships and social support embedded within systems (e.g., families, schools, and communities) [11, 12]. Resilience theory provides a conceptual framework for considering a strengths-based approach to understanding why some children and adolescents of color living in poverty ‘beat the odds’ and grow up to be productive well rounded healthy adults [10]. Resilience models propose relationships and processes that strengthen protective factors and inoculate youth from risks associated with living in poverty [10, 12].

Protective factors are adaptive systems that account for a significant amount of the capacity of a child’s ability to adapt to challenges and adversities encountered as they grow up in families, communities, and society at large [11, 12]. These protective factors include assets that reside within an individual, such as self-efficacy, self-esteem, competence, motivation, assertiveness, and spirituality as well as resources within their context (e.g., parental support, extended family, adult mentors, churches, and youth programs) [11, 12]. While some studies of resilience models have found that protective factors interact with risk exposures to produce outcomes, little has been done to determine why this is so. For example, if a study reports that parental support (a protective factor) interacts with negative peer influence (a risk) to prevent alcohol abuse (an outcome), the next step should be to understand how parental support defuses the negative peer influence. Parents may provide the emotional support necessary to withstand peer influence or may provide informational support related to the health consequences of unhealthy behaviors such as alcohol, smoking, and drug usage [13, 14].

Recent studies have shown that children are capable of expressing valuable perspectives about their lived experiences [15–17]. This study is child centered in that a draw-and-write technique was used as a way of inviting children between the ages of 9 to 14 years, to make visual representations of the resources that, in their view, help them make it in life. By not imposing any kind of structure or expectation, and by listening to what children have to say – in this case, seeing what they have to draw – grants precedence to children’s experience and respect to their personhood. In addition to the draw-and-write, in depth interviews were conducted with a sub-set (15) of the children who agreed to discuss the meaning of the images in their drawings.

The primary goal of this study was to identify the protective factors and processes that promote resilience from the child’s point of view. A summative content qualitative analysis [18, 19] based on a resilience framework was used to determine the feasibility of a draw-and-write technique in exploring protective factors that promote resilience in youth of color living in poverty [20]. Once identified, these factors could be the target of future interventions designed to produce resilience in youth of color living in poverty.

## Methods

### Site

Two low-income housing apartment buildings located in an urban community in the Midwest were selected for the study. Each housing site has a community learning center (CLC) which is equipped with desks, computers, and school supplies. All of the families whose children participated in this study were residents of low-income housing who faced risks associated with living in poverty. However, while most Americans live in neighborhoods among people of their own race or ethnicity, residents of these low-income housing apartments have the privilege of living in an ethnically and racially diverse community.

While the children enrolled in the CLC encountered risks associated with living in poverty, they shared several characteristics associated with resilient outcomes: 1) good academic standing; 2) strong social ties (e.g., good relationships with parents, teachers, peers and CLC staff); and 3) active engagement in youth organizations. This study provided an opportunity to uncover similarities and differences in the resources and relationships that children from different racial/ethnic backgrounds say helps them to “chase their dreams” or make it in life.

### Recruitment

After receiving approval from the University of Wisconsin-Madison Social Sciences Human Subjects Protection Committee (2018-1161-CP001), the CLC managers met with the principal investigator (PI) to discuss recruitment strategy and agreed upon the following inclusion criteria: 1) children between the ages of 9-14 years who participate in the afterschool program at the CLC, and 2) plan to reside at the housing facility for at least one year. The CLC managers invited all children from their afterschool program who met the inclusion criteria to participate in the project. We held an evening dinner for 36 families from the CLCs and explained the study in detail using the University of Wisconsin-Madison Social Sciences Human Subjects Protection Committee consent and assent forms as a reference. The parents and children were asked to read the consent and assent form and were provided an opportunity to ask questions about the study. Informed assent and consent was obtained from 33 subjects ages 9 to 14 years (mean age 12.4 years) and their legal guardians. Three parents declined because they were planning to move out of the housing unit within a year. The convenience sample included 23 females and 10 males, of which 10 were Hmong (7 females and 3 males), 11 were Middle Eastern (8 females and 3 males), and 12 were African American (8 females and 4 males). At the completion of the draw and write, 15 of these children (4 Hmong, 4 Middle Eastern, and 7 African American) agreed to be interviewed. All the children attended public schools and were from families with annual incomes less than 200% of the federal poverty level.

### Data collection and analysis

We held three draw-and-write activity sessions. Each session included eleven participants. The participants were seated in such a way that they could not see what the others were drawing. Each child was provided with a pencil and paper and was asked, “Can you draw a picture of a person or thing that has helped you to make it this far in your life?” The children were also asked to write a brief description of the image they drew. After completing the pictures, all the children were invited to participate in a one-on-one non-directional interview with the PI. Fifteen children agreed to be interviewed. The interviews were scheduled within seven days of completing the picture. We began the interview by asking the child to describe their picture, what the objects in the picture meant to them, and how the objects have helped them do well in life so far.

### Analyses of drawings and interviews

A summative content analysis of the data allowed both a qualitative assessment of what was drawn and quantitative consideration of how often particular themes or categories appeared, and whether these patterns differed based on other factors such as gender and ethnicity [18, 19]. A resiliency framework was used to identify factors that may have supported positive outcomes despite risks associated with low-income and/or marginalized minority status. The PI read each narrative, and transcript and reviewed each drawing carefully before highlighting text that appeared to describe a person, place or thing that helped a child make it in life. A key word, or phrase that seemed to capture the child’s perspective was written in the margin of the text, using the participant’s words. After inductively analyzing one third of the drawings, written narratives, and transcripts, the PI decided on preliminary codes. For example, when a participant drew a school building and school paraphernalia and explained that school and education enabled them to make it in life by offering a pathway to chase dreams, it was labelled “education supports future aspirations”. The preliminary coding scheme which consisted of eight codes (teacher supports educational activities, parents support, friends (peers) support, Mom protects/advocates, Mom supports educational activities, sibling helps, parents supports education, and Dad supports) was used to deductively analyze the remaining drawings, narratives, and transcripts and new codes were added when data did not fit into the preliminary coding scheme.

**Fig. 1:**
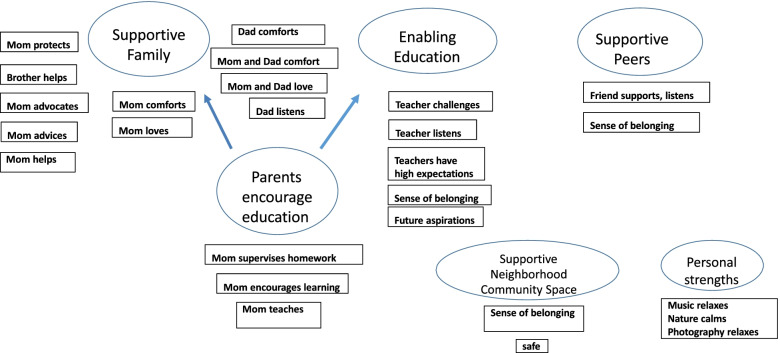
Category mapping

Once all transcripts and drawings were coded, the data within each code was examined again. Codes with similar content or context were collated into categories labelled protective factors. For example, the codes mom supervises homework, mother encourages learning, mom helps, and mom teaches were grouped under the category parent encourages education (Fig. 1). This resulted in six protective factor categories: supportive school, supportive family, parents encourage education, positive peers, supportive community space and personal strengths. The frequency of each protective factor category was recorded. In the results, the protective factors were described using the identified codes and categories. In the discussion of the findings, the results from this summative content analysis was compared with other studies to highlight similarities and differences.

## Results

**Fig. 2:**
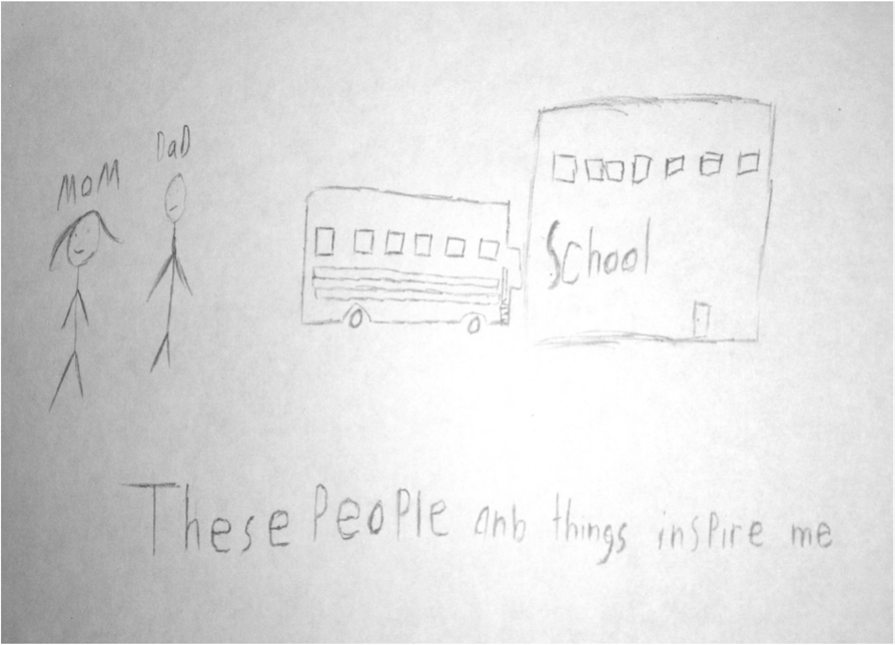
Parents and school inspire

In this study 33 participants indicated that they “were able to make it so far” because they had active support systems. Two drawings described personal strengths as a protective factor. Therefore it was added to the category list since it captured something important in relation to the research question. The summary of categories mentioned in Table 1 refers to the percentage (%) of participating youth whose data were coded within a category (Table 1). Fourteen of the 33 participants data were coded in more than one category (Fig. 2).

**Table 1:** Protective factor categories

**Categories**	**% of Children *n=*33**
Supportive Family	82% *n=*27
Parents Encourage Education	27% *n=*9
Supportive Teachers	12% *n=*4
Supportive Peers	12% *n=*4
Supportive Neighborhood and Community	6% *n=*2
Personal Strengths	6% *n=*2

The draw-and-write activity enabled the researcher to document the protective factors that ”helped these children make it so far”. The categories identified in the analysis were predominately descriptive, i.e. they described patterns in the data relevant to the research question. The following categories were generated in the written narratives and interviews: supportive family (e.g.,caring parent(s) or siblings); parents encouraging education; supportive teachers; supportive peers; supportive neighborhood and community; and personal strengths (e.g., expressive skills like relaxation, music, drawing and photography, Table 1).

### Supportive family

Twenty-seven drawings were categorized as family support. Nine children drew photos of both parents, two children drew a picture of their father, and one drew a picture of a sibling. Fifteen participants identified their mother as a supportive person in their lives.

**Fig. 3:**
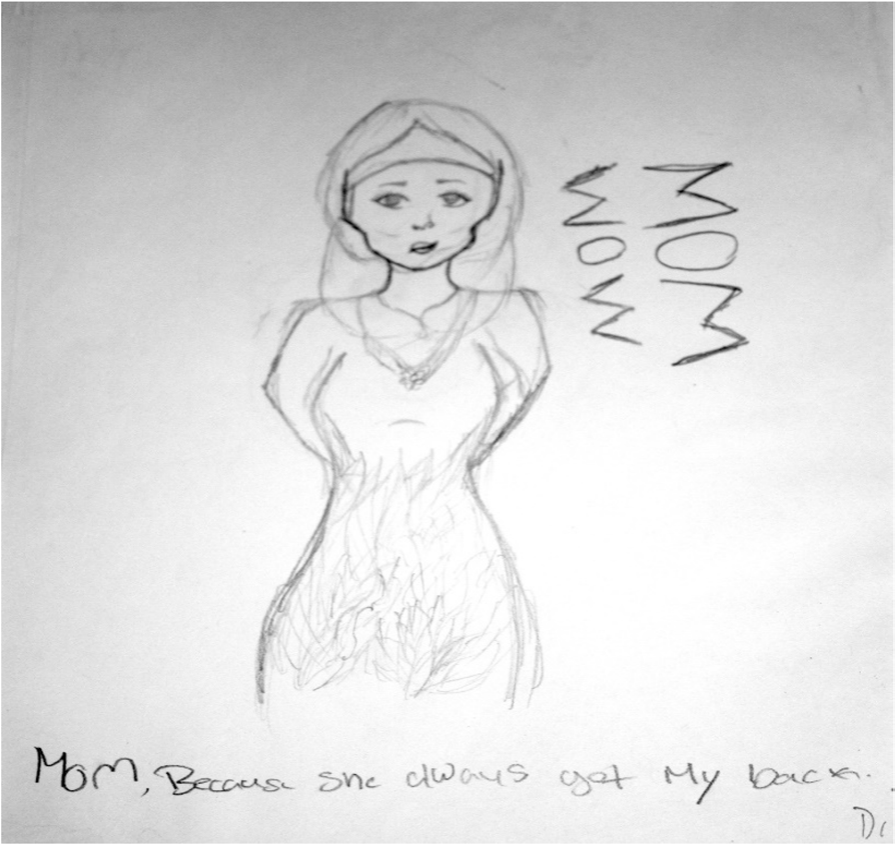
Mom is my best friend

Girl D, a 13-year-old Middle Eastern student stated in response to the question *“My mom because she always has my back”* (Fig. 3). Girl D stated the following during her interview*:” My Mom is my best friend. She buys me lots of stuff like clothes, jewelry-stuff we couldn’t get in Iran. When I am in trouble or sad I go to my Mom. If I need help with anything I would go to my Mom.”* During an interview Girl R, a 13 year old Middle Eastern participant stated in response to the question, *“My mom helped me by talking to me about stuff. She is like my therapy.”*

Resilience came up during the interview with Girl N, a 13 year old African American participant who stated in response to the question the following, *“I drew a picture of my mom because my mom is the most important person in my life. And she is the one who helps me every day to get through the bad things at school and stuff. He (her father) call me names like dumb, ugly and stupid, but I just go to my mom and she talks to me about it and says like so don’t talk to him and stuff. He is mean. He always fights with my brother. One time the police took him away for fighting my brother.”*

Although the focus was on factors that helped children, sometimes discussions of risk came up. School was a risk factor for Girl N, because she was bullied by peers**. *“****Everybody like tortured me. Like there was like a whole group of people just swarming me and telling me all these bad stuff about myself. They (teachers) really didn’t do anything anyways. They said that they were going to tell somebody. But they didn’t tell anybody.”*

Resilience emerged for Girl N because her mother was her advocate and spoke out on her behalf. Girl N said her mother went to the school regularly to find ways to create a supportive environment for her. At her mother’s request, Girl N was moved to another classroom. She no longer experiences bullying with her new classmates. Girl N indicated that her grades improved and she has made new friends. Girl N stated *“My mother is my hero.”*

While mothers were identified as a strong source of support, a few participants drew pictures of their father. Boy M, an 11 year old Hmong participant drew a picture of his dad and wrote the following in his narrative, *“My dad always calms me down if I am sad or get bullied or something. He is always helpful”.* Boy A, an 11-year-old Middle Eastern participant wrote in his written narrative, *“my brother helps me when I need help.”*

#### Parents encourage education

**Fig. 4:**
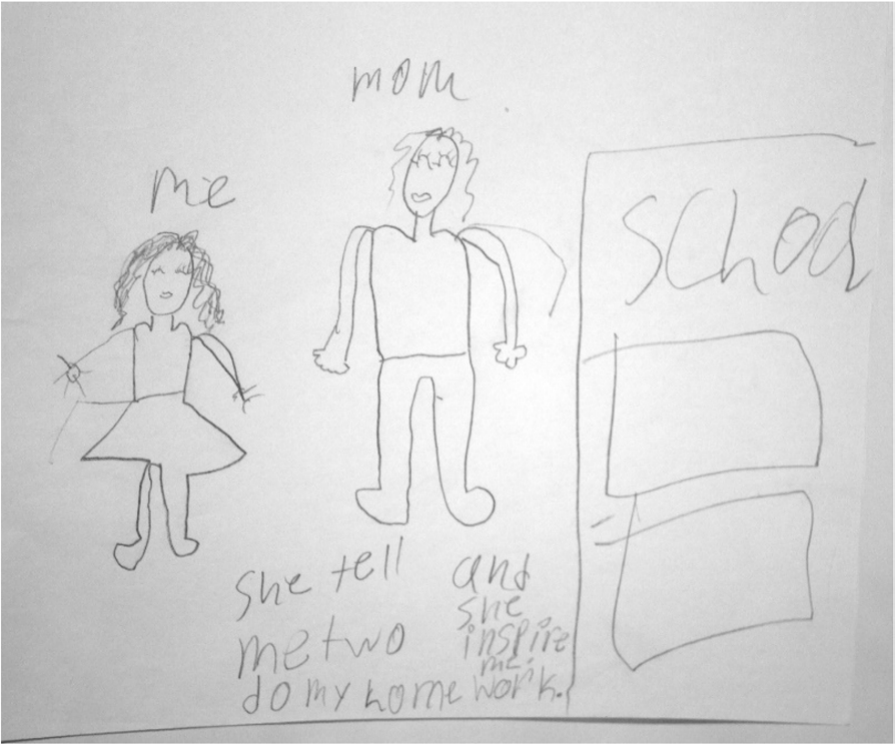
Mom encourages education

 The written narrative of nine participants was coded parents encouraging education. These children drew pictures of parent(s) and a school building. Seven of the nine pictures were drawn by African American children. Girl K, a 10 year old African American participant wrote in her narrative that her mother inspires her and “*tells me two (sic) do my homework”* (Fig. 4). Girl D, an 11 year old African American participant stated during an interview, *“my mother wants me to have a better life than her. She said the only way to be a success is by going to school every day.”* Girl S, a 10 year old Hmong participant wrote in her narrative, *“My mom helps me with my homework.”*

#### Supportive teachers

**Fig. 5:**
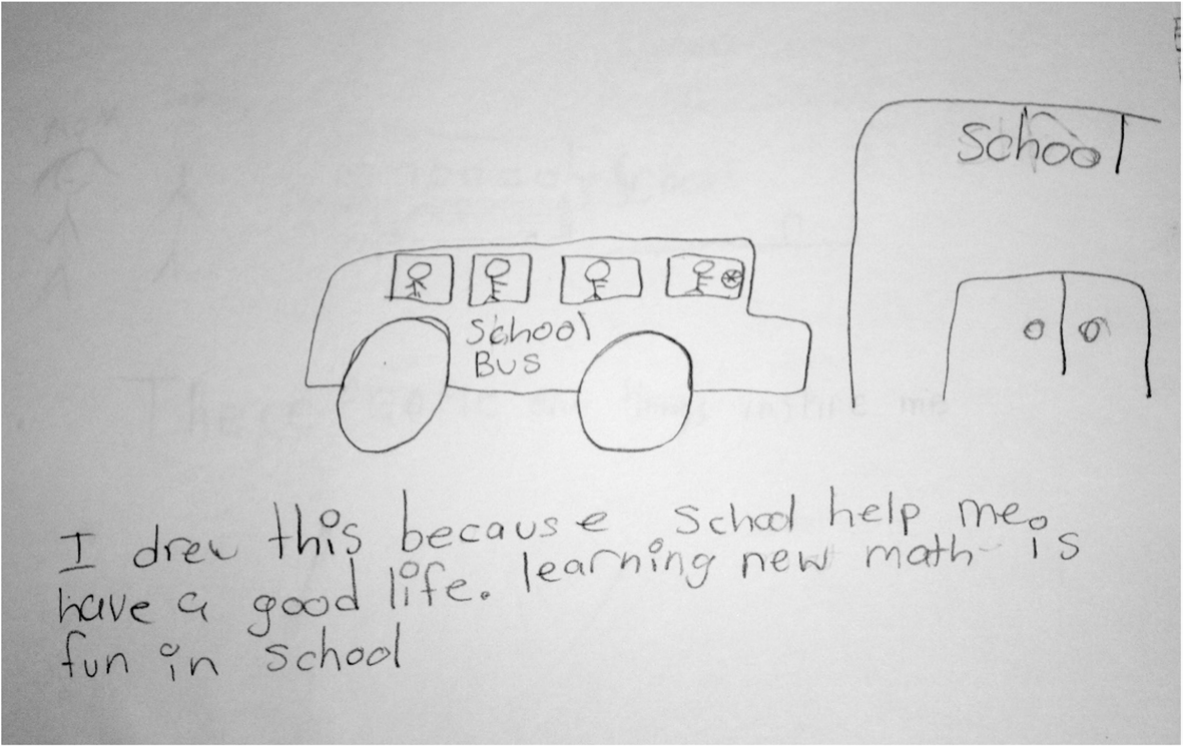
Education allows me to “chase my dreams”

Four participants mentioned in their written narrative that having access to caring and supportive teachers enables resilience. Boy T, a 12 African American participant drew a picture of a school bus and a school building (Fig. 5). He wrote in his narrative the following, *“I drew this because school help me have a good life.”*

*Learning new math is fun in school.”* T stated during his interview *tha,t “an education would allow me to chase my dreams.”* T aspires to be a basketball player or construction worker. He really enjoys math, but dislikes reading. He described his teacher as being very helpful, willing to listen and supportive.

Girl D, a 13 year old Middle Eastern participant stated the following during her interview, *“In Iran, school wasn’t fun. They didn’t have any fun things for us to do. I really like school here in the Midwest. I have a lot of friends. I want to do good in school so I can take care of my parents when I grow up.”* Boy J, a 12-year-old Hmong participant wrote the following narrative, *“My math teacher helps me be successful by helping me with math.”* Girl C, a 13-year-old Hmong participant stated, *“I want to do well in school so I can become a surgeon when I grow up.”*

#### Supportive peers

Four participants reported meaningful connections to peers at school. Girl O, an 11-year-old African American participant wrote in her narrative, *“I go to them (friends) for everything and we are very close and I love them so much.”* Girl D, an 11-year-old Middle Eastern participant wrote the following, *“I drew my friends because they always help me in hard times;”*

#### Supportive neighborhood and community

Two children drew an image of the CLC, identifying it as a ‘safe place’ to hang out with friends and work on homework. Girl R, a 10-year-old African American participant stated during her interview, *“My home has helped me because we don’t have to worry about living in a car anymore. It is the place where I feel the most comfortable in. There are lots of people around us that care about us. I love my neighbors and the people at the CLC. Everyone is nice to us. I have lots of friends here.”* Girl I, a 13 year old Hmong participant drew a picture of the CLC building stating, “*My parents help me make it this far from putting me in programs like this one or others.”*

#### Personal strengths

**Fig. 6:**
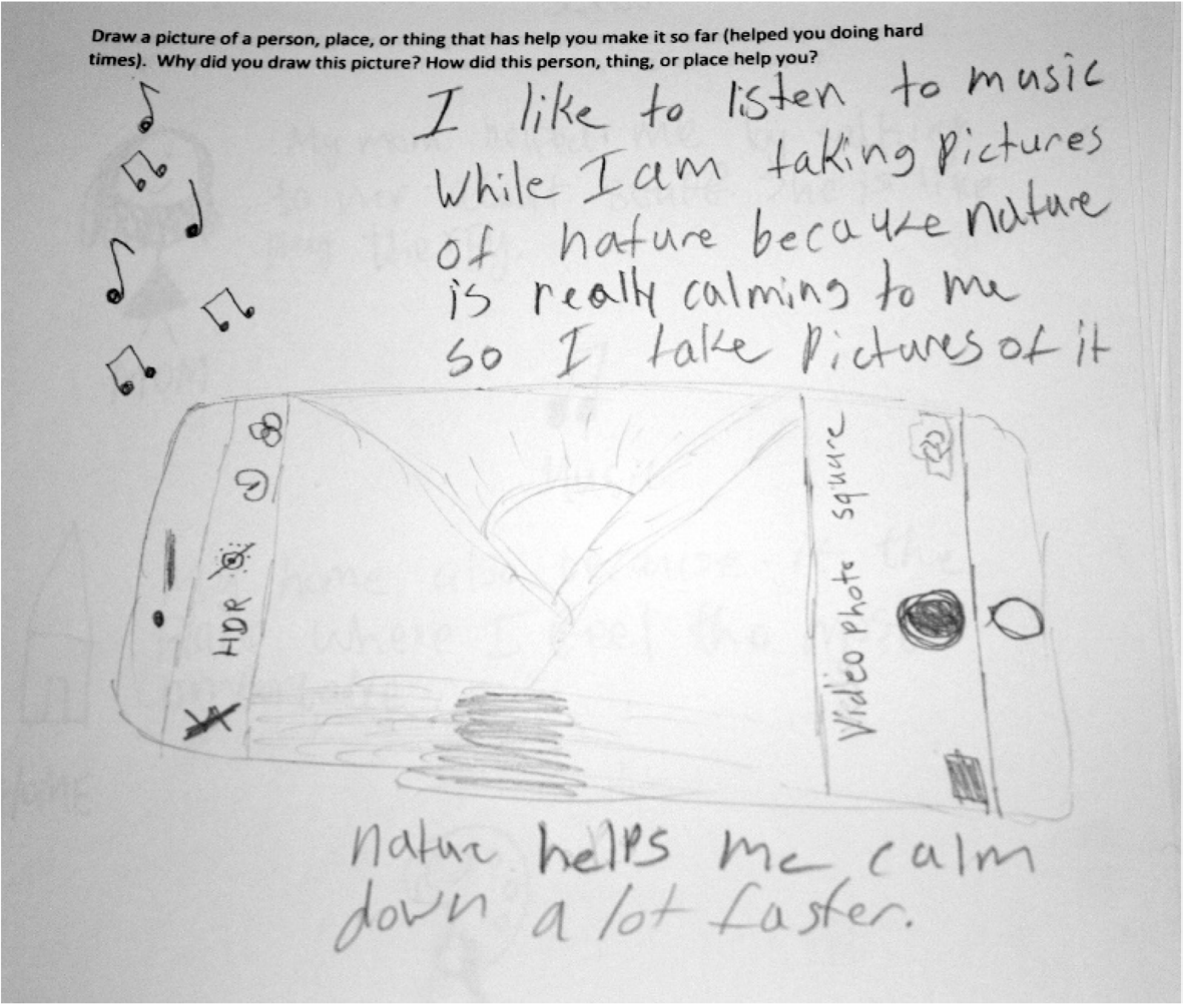
Music calms

One of the personal assets that enabled the resilience of the participants was relieving stress and anger through music and photography. Girl M, a 13 year old Middle Eastern participant drew a picture of a cell phone (Fig. 6).

She wrote in her narrative*, “I like to listen to music while I am taking pictures of nature because nature is really calming to me. So I take pictures of it. Nature helps me calm down a lot faster.”* Girl C, a 13 year old Hmong participant wrote in her *narrative, “I listen to music when I’m not in the mood to talk to people, like being mad. I would listen to music because it calms me down.”*

**Fig. 7:**
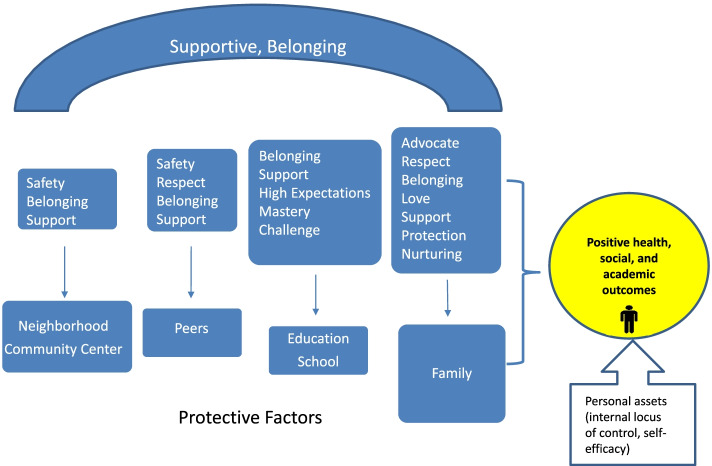
Attributes of protective factors

The use of the draw-and-write technique enabled the documentation of the protective factors that children perceived to help them make it in life. These protective factors included family members, (particularly the mother), supportive relationships at school, personal assets, and community centers as safe spaces. The participants also described the attributes of the protective factors which helped them make it in thus far in life. For example, one participant described her mother as her hero because her mother provides emotional support when she is troubled or sad. The attributes of each resource as described by the participants are listed in Fig. 7.

## Discussion

This study explored children’s perspectives about the protective factors that help them rise above the numerous adversities and obstacles they face growing up. Studies involving vulnerable youth have shown that children are more likely to be resilient if their social ecologies are supportive and nurturing [21–23]. My preliminary findings support existing literature that resilience of a child depends on resilience of interconnected systems, particularly families and schools [10, 14, 24]. These findings were consistent across ethnicity and gender.

Most of the participants in this study described their parent(s) as nurturing and supportive, enabling them to overcome obstacles and adversities within their environment. “Parents and other caregivers who are able to form close, nurturing relationships with their children can foster resilience in them that protects them from many of the worst effects of a harsh early environment” [25]. Developmental research also supports the mitigating effects of family cohesion, nurturing, and stable relationships on the negative outcomes of early adversity [26]. For example, Girl N indicated that she was able to endure verbal abuse from her father because of the emotional support and compassion she received from her mother. Girl N admitted to engaging in self-harm behavior (i.e., cutting) because of exposure to turmoil within her home environment and ongoing bullying at school. Her mother advocated on her behalf with the school system to ensure she was placed in a classroom setting with a caring teacher and peers who valued education and provided a sense of belonging. As a result, Girl N’s grades improved and she developed new friendships among her classmates. Her mother also sought professional care for her daughter’s mental distress. Girl N stated that her therapist helped her manage her emotions and behavior when she was feeling distressed or overwhelmed. She no longer engages in self-harm behavior and engages in positive coping skills (e.g., seeking help from mother or teacher, drawing and paintings, and physical activity). Girl N is an example of how a protective factor (mother’s support) interacted with risks (unsupportive father and bullying from peers at school) to result in a resilient outcome (improvement in grades and new friends). Interventions that empower African American parents to advocate for their children as in the case of Girl N are sorely needed. Structural racism influenced laws and policies that created adverse living conditions for African Americans and other people of color [27]. Residential segregation continues to restrict many African Americans’ access to quality education and desirable employment opportunities, thus limiting opportunities to escape poverty [28]. As a result, intergenerational poverty is more prevalent in African Americans than any other racial or ethnic group in the United States [1, 29]. The key to breaking the intergenerational cycle of poverty for African Americans is access to quality education that opens the gateway to higher paying jobs [30]. Girl D, an 11 year old African American participant stated during an interview, *“My mother wants me to have a better life than her. She said the only way to be a success is by going to school every day.”* Williams and Bryan found that African American students from low-income, single-mother-led households who received verbal praise for good grades, supervision and help with homework, and monitoring of academic performance in school were more likely to be successful academically [21]. The seven African American participants who drew pictures of a parent and a school building reported that their mothers were very supportive and emphasized education as a means to escape poverty.

A school environment that provides a sense of belonging has a positive impact on student achievement [2, 31]. T Williams and Bryan reported specific factors at school that contributed to improved academic engagement and performance [21]. These factors included: “(a) having at least one caring adult at the school (e.g., a teacher, counselor, coach, or college recruiter) who displayed warmth, concern, openness, and understanding; (b) the importance of close friendships among peers who valued education, despite similar negative circumstances” [16]. Most of the participants reported a sense of belonging at their school and indicated that their teachers were supportive. Boy T stated during his interview *that, “An education would allow me to chase my dreams.”* Boy T described his teacher as being very helpful, willing to listen and supportive. This supportive relationship encouraged Boy T to dream of becoming a basketball player or construction worker.

Like families and schools, communities can provide relationships that support resilience in youth. Bernard comments that “communities exert not only a direct influence on the lives of youth but, perhaps even more importantly, exert a profound influence on the lives of the families and schools within their domain and, thus, indirectly powerfully affect the outcome for children and youth” [31]. Neighborhoods that foster resilience in youth have some if not all of the following features: “(a) safe recreational facilities, (b) educational and employment opportunities, (c) preventative health care (d) adult mentors and counselors (e) well-developed and integrated networks of social organizations (f) school-based community services, and (f) available religious communities” [27]. Youth who engage in school and community-wide activities that promote expressed social and cultural norms and a high expectation for good citizenship tend to excel in the academic setting [2, 31]. Girl I, a 13 year old Hmong participant drew a picture of the CLC building stating, “*My parents help me make it this far from putting me in programs like this one or others.”* The CLC was a safe recreational facility that provided tutoring for children and offered fun activities. All of the children enrolled in the CLC were on good academic standing and were engaged in community activities.

Emotional awareness, the process of identifying when one is stressed or angry is an important part of the resilience process. Finding an internal locus of control where one feels they can control how they feel by engaging in healthy coping strategies can have a positive impact on mental health [32]. The draw-and-write technique was effective in eliciting coping strategies used by participants for stress and anger, namely music, nature, and photography. Other research has emphasized the benefits of these coping strategies, for example, a recent study reported that nature has a positive impact on children’s health by promoting opportunities to be physically active, play, and enjoy viewing plants and animals [33].

This draw-and-write activity was a successful way to explore the youths’ perspectives on relationships and processes that help them rise above the numerous adversities and obstacles they face growing up in their environment. A strength of this study was our ability to gain the perspective of children from different racial/ethnic groups living in poverty. While several studies provide an account of factors that influence the health of African American children living in poverty, little is available about Hmong or Middle Eastern children [20]. In this study African American, Hmong, and Middle Eastern children identified parent(s) as having a strong influence on their success in life. In this sense, regardless of racial/ethnic background, parents, and especially mothers, were the main “person or thing” identified by these children living in poverty as helping them “make it thus far in life.” Forty five percent of participants identified their mother as a key anchor in their life. Fifty eight percent of the African American children indicated that their parent(s) encouraged education as a means to escape poverty. The Hmong and Middle Eastern participants also indicated that an education would help them obtain a good job when they grow up. Thus, although education was mentioned by all groups, there were cultural differences in the way in which children perceived of education.

Although the multiple ethnic backgrounds of the study offer unique insights, it is important also to note a limitation of this study. The children were all drawn from a single site. Children who had behavioral/social problems were not allowed to use the facility limiting the ability to obtain the perspective of children with fewer support systems. It will be important for future work to explore these issues in a range of settings and continue to seek insights from a full range of children.

The results of this study support the findings of other studies that use a resilience approach [15, 16, 20, 34, 35]. In spite of losing their parents to AIDs and related illnesses, the participants in a draw and write study conducted in South Africa identified the personal strength of “having dreams for the future” [20]. This overarching theme of a “dream for the future” is similar to our participants’ views of “chasing a dream” which engenders hope and a desire to be successfully in spite of adversities faced.

## Conclusion

The initial interviews, observations, and the draw and write pictures of 33 youth suggest that families, particularly parents, have the strongest influence on a child living in poverty. If the home environment is nurturing and supportive, internal assets such as confidence, self-esteem, and coping skills are strengthened, enabling a child to overcome obstacles and adversities within his or her environment. Social resources such as schools, teachers and peers also contribute to the resilience process if they provide support and a sense of belonging.

Interventions that target interconnected systems will contribute to the process of resilience and are solely needed. For example, an intervention that is designed to empower single mothers to advocate for their children who attend an under resourced school (risk factor) may increase the competence and self-efficacy of a child (assets) as evidenced by a positive outcome (academic achievement). The participants of this study who were approaching adolescence appeared to have strong supportive systems that will increase the likelihood that they will navigate the risk associated with adolescence provided they maintain strong ties with their family and other protective factors within their environment.

Unlike Girl N’s mother, some African American mothers may not have the confidence or skills to advocate for their child. Mothers living in poverty often experience social isolation and decreased access to resources [12, 13, 24]. This can lead to disturbances in the mental or physical health of a parent that can disrupt the quality of care provided to the child. Social support interventions targeting low-income at-risk families are needed counteract many of the negative effects of early adversity and intergenerational poverty [12, 13]. Since resilience is embedded in relationships and social support, interventions that strengthen parenting skills and parent-child relationships in settings that parents already access (e.g., healthcare offices, community and faith-based organizations, schools, and homes), have the potential to have a significant impact on children’s health and developmental outcomes [24].

## Data Availability

(data transparency)- The datasets used and/or analyzed during the current study are available from the corresponding author on reasonable request.
